# Core outcomes set for research on the treatment of opioid use disorder (COS-OUD): the National Institute on Drug Abuse Clinical Trials Network protocol for an e-Delphi consensus study

**DOI:** 10.1186/s13063-021-05051-9

**Published:** 2021-01-28

**Authors:** Niranjan S. Karnik, Cynthia I. Campbell, Megan E. Curtis, David A. Fiellin, Udi Ghitza, Kathryn Hefner, Yih-Ing Hser, R. Kathryn McHugh, Sean M. Murphy, Sterling M. McPherson, Landhing Moran, Larissa J. Mooney, Li-Tzy Wu, Dikla Shmueli-Blumberg, Matisyahu Shulman, Robert P. Schwartz, Kari A. Stephens, Katherine E. Watkins, John Marsden

**Affiliations:** 1 Department of Psychiatry & Behavioral Sciences, Rush University Medical Center, 1645 West Jackson Blvd., Suite 600, Chicago, IL 60612 USA; 2 Division of Research, Kaiser Permanente Northern California, 2000 Broadway, Oakland, CA 94612 USA; 3 Department of Psychiatry and Biobehavioral Sciences, University of California, Los Angeles, 11075 Santa Monica Blvd., Suite 200, Los Angeles, CA 90025 USA; 4 Yale School of Medicine, Internal Medicine, Program in Addiction Medicine, PO Box 208056, 333 Cedar Street, New Haven, CT 06520-8056 USA; 5 National Institute on Drug Abuse, National Institutes of Health, National Institute on Drug Abuse Center for Clinical Trials Network, 6001 Executive Blvd, Bethesda, MD 20892 USA; 6 The Emmes Company, LLC, National Institute on Drug Abuse Data and Statistics Center and Clinical Coordinating Center, 401 N Washington St, Rockville, MD 20850 USA; 7 Division of Alcohol, Drugs and Addiction, McLean Hospital, & Department of Psychiatry, Harvard Medical School, McLean Hospital, Proctor House 3, 115 Mill St, Belmont, MA 02478 USA; 8 Department of Population Health Sciences, Weill Cornell Medical College, 1300 York Avenue, New York, NY 10065 USA; 9 Washington State University Elson S. Floyd College of Medicine, 412 E. Spokane Falls Blvd., Spokane, WA 99202-2131 USA; 10 Duke University School of Medicine, Department of Psychiatry and Behavioral Sciences, Duke University Medical Center, Box 3903, Durham, NC 27710 USA; 11 Department of Psychiatry, Columbia University Irving Medical Center & Department of Psychiatry, New York State Psychiatric Institute, 1051 Riverside Dr., New York, NY USA; 12 Friends Research Institute, 1040 Park Avenue, Suite 103, Baltimore, MD 21201-5633 USA; 13 Departments of Family Medicine, Biomedical Informatics & Medical Education, University of Washington, Seattle, WA 98195 USA; 14 RAND Corporation, 1776 Main Street, Santa Monica, CA 90401-3208 USA; 15 Addictions Department, Division of Academic Psychiatry, Institute of Psychiatry, Psychology and Neuroscience, King’s College London, DeCrespigny Park, Denmark Hill, London, SE5 8AF UK

**Keywords:** Core outcome set, Opioid use disorder, Patient-reported outcomes, E-Delphi survey, Outcome reporting, Consensus

## Abstract

**Background:**

A lack of consensus on the optimal outcome measures to assess the efficacy and effectiveness of interventions for the treatment of opioid use disorder (OUD) has hampered the pooling of research data for evidence synthesis and clinical guidelines. A core outcome set (COS) is a minimum set of outcome measures that are recommended for all studies of a particular condition. The National Drug Abuse Treatment Clinical Trials Network (CTN) Core Outcome Set for OUD (COS-OUD) is a development study to identify core constructs, meaningful outcomes, and their optimal measurement for all efficacy and effectiveness studies of OUD treatment and service delivery.

**Methods/design:**

Overseen by an expert workgroup, a modified, stepwise, e-Delphi methodology will be used to gain consensus among a panel of clinical practitioners and researchers involved in the treatment of OUD, who are members of the CTN. Sequential rounds of anonymous, online questionnaires will be used to identify, rate the importance of, and refine a core outcome set. A consensus threshold will be achieved if at least 70% of the panel rate the measure as critical for inclusion in the COS-OUD. Where consensus is not reached or there are suggestions for new measures, these will be brought forward to a further round of review prior to a consensus meeting. Products from this study will be communicated via peer-reviewed scientific journals and conferences.

**Discussion:**

This initiative will develop a COS for OUD intervention trials, treatment studies, and service delivery and will support the pooling of research and clinical practice data and efforts to develop measurement-based care within the OUD treatment cascade.

**Trial registration:**

http://www.comet-initiative.org/Studies/Details/1579

## Background

Opioid use disorder (OUD) is a debilitating biobehavioral condition [1] with major social overlays [2] that is associated with a substantial global burden of disease [3] and, in several countries, a sharp recent increase in overdose fatalities [3, 4].

To address the opioid epidemic, ongoing international research efforts aim to create innovations with front-line opioid agonist, partial agonist/antagonist, and antagonist therapies for OUD. These include new developments of extended-release formulations of some medicines, evaluations of adjunctive psychosocial interventions, and applied efforts to expand patient access in priority healthcare settings [5].

In the past four decades, evidence for the efficacy of the medication and psychosocial treatments for OUD has accumulated from randomized controlled trials and observational studies using one or more of the following direct or indirect assessments of benefit: patient-reported outcomes (PRO), clinician-reported (ClinRO), observer-reported (ObsRO), or performance outcomes (PerfO). Measures have included self-reported drug use, problem checklists, personal functioning, biomarkers, and indicators of treatment retention and completion. However, no consensus exists on the optimal ways of measuring clinical benefit and other patient outcomes. Accordingly, clinical trials use a wide array of measures and operational definitions of key outcomes, limiting the ability to make direct comparisons across studies, to aggregate data across studies, and to allow for benchmarking.

Established in 1999, the National Drug Abuse Treatment Clinical Trials Network (CTN) is a network of university- and health system-based research groups and healthcare service providers that collaborate on research on OUD and other substance use disorders, and coordinates data and discovery across a multitude of trials. De-identified research data from these completed trials are distributed to the scientific community and the public at large through an electronic repository, the National Institute on Drug Abuse Data Share website [6, 7]. One feature of the website is that it displays the assessments used in each study, by construct-based category, and identifies studies that use similar assessments. This, in part, is meant to encourage the use of common instruments in future studies examining similar constructs (e.g., depression).

In 2000, in a more targeted effort of harmonization, the CTN recommended a common assessment battery for research [8]. This was subsequently included in 21 randomized controlled studies, but feedback from patients, service providers, and researchers concluded that the battery was administratively burdensome and infeasible in practice, largely because of the number of items [9]. In 2009, a CTN taskforce recommended a shorter set of instruments [10], but these were not widely or consistently adopted [8]. Interestingly, this task force did find a desire to have a “minimum (i.e., core) data set as a standard” S2: [11]. In 2014, a public portal was launched providing access to 18 recommended screening and clinical research instruments [12], with integration support for inclusion in clinical research and electronic health records (EHR) [13, 14]. In 2019, the Data & Statistics Center of the CTN, under commission from the CTN Steering Committee, developed and published a battery of standardized assessments for research [15]. The battery includes standardized forms for data gathering, measures that have been extracted from the NIH PhenX toolkit [16], and many other validated tools that have high relevance to drug misuse research. Overall, these prior initiatives produced highly valuable syntheses of tools and instruments, but their overall length and number of items prevent universal use as a COS.

In the era of measurement- and value-based care, key stakeholders have an increasing expectation that health care service providers incorporate standardized and clinically actionable measures in their EHRs to facilitate data pooling in healthcare systems. Several ongoing initiatives have this goal in mind, including the Patient-Reported Outcomes Measurement Information System (PROMIS), which includes over 300 measures organized in profile domains; the consensus measures for phenotypes and exposures initiative which includes approximately 800 measurement protocols in toolkits; and the Fast Healthcare Interoperability Resource (FHIR) standard for healthcare data exchange produced by the Health Level 7 international community. In a series of workshops and publications, the Food and Drug Administration has promoted clinical outcome assessments and called for greater use of patient-reported outcomes in research studies [17]. NIDA and other federal agencies have also sought evidence of the benefits of decreased substance use short of abstinence.

In spite of these sustained and multiple ongoing efforts, implementing common measures for OUD treatment studies has been elusive. A solution to the problem of outcome measurement consensus that has gained success in many areas of medicine is to identify the absolute minimum set of measures—the so-called core outcome set (COS)—that are to be reported in all efficacy research studies on a target condition [18]. A COS does not restrict outcome measures, and it is expected that researchers would usually need other measures to address specific study aims, but it is expected that using a COS will result in studies of higher quality, applicability, and impact. This approach provides researchers with the needed flexibility to target assessment batteries to their study aims and population of interest, while maintaining the benefits of a common assessment battery to facilitate comparison across studies and the aggregation of datasets. The Core Outcomes Measures in Effectiveness Trials (COMET) database lists preregistered COS development (http://www.comet-initiative.org).

Most COS development studies use a Delphi methodology. First developed by the RAND Corporation in the 1950s and 1960s, Delphi is a family of participatory and stepwise procedures for eliciting and refining the opinions of a panel of people who are usually experts on a particular topic. The panel consents to have their views and value judgments gathered and combined to establish a consensus on a topic of complexity where there is uncertainty, and where there are concerns that current practice or policy is an impediment to progress [19, 20]. Delphi studies follow four principles: anonymity of panel members’ responses (to mitigate against group pressure and the biasing influence of dominant viewpoints), iteration, controlled feedback, and statistical aggregation of panel responses [21].

Commonly, COS development studies proceed through four steps. In the first step, a concept mapping of the topic is done by a study workgroup to set the scope. A second step involves a systematic review of existing outcome measures to gather potentially relevant material. The third phase is a sequence of questionnaires (usually web-based) sent to the panel, beginning with an open-ended format to canvass a wide range of outcome options and progressing through a closed format with instructions to estimate the relevance and importance of potential measures. Removal and modifications of measures in successive rounds are done according to a predetermined criterion alongside opinion feedback. Further rounds of questionnaires are issued until a consensus is judged to have been reached by the achievement of the pre-determined agreement threshold. Finally, a consensus meeting is held to discuss the study products and resolve outstanding issues, prior to the communication of results. On completion of these four steps, results are publicized.

The need for consensus on outcome measurement for OUD research has intensified in the context of the current opioid crisis and the large number of planned OUD-related research studies at the CTN supported by the National Institutes of Health’s Helping to End Addiction Long-term (HEAL) initiative [22]. In March 2020, with advice from the Center for Clinical Trials Network staff and leadership, a proposal was submitted to the CTN Research and Development Committee Great Lakes Node to lead a COS development study on OUD-related research. At the March 2020 CTN Steering Committee Meeting Webinar, a workgroup was formed to develop the protocol and implement the COS-OUD study. The co-authors of this manuscript constitute the workgroup. This workgroup determined the protocol for the establishment of the eDelphi panel. A subset of the workgroup also participates on the panel along with a broader group of stakeholders from within the CTN.

## Aims and research questions

With only one prior OUD study registered on the COMET database, this article describes a different e-Delphi protocol for COS-OUD which has different goals than COMET protocol 1128 [23]. The goals of this study are to identify a COS for OUD-related treatment efficacy and effectiveness research; to develop guidelines for their recording, analysis, interpretation, and communication; and to promote their use for research and clinical practice. Products from COS-OUD are also envisaged to contribute to efforts to develop measurement-based care for OUD interventions [24]. The study will address the following four questions:

1. What are the core constructs or domains to be included in the COS?
2. What are the differences in priority and perspective between different professions?
3. What outcome items are rated most important?
4. How should outcome items be optimally defined and captured?

COMET protocol 1128 which was funded by the Canadian Institutes of Health Research in March 2018 has produced systematic reviews [25, 26], but the protocol has not produced a defined outcome set. In addition, their goal is much larger in trying to develop a comprehensive set of patient-oriented outcomes. Our aim is to establish a minimum set of key outcomes that can be used for study linkage across the OUD treatment cascade.

The COS-OUD will comprehensively cover adult OUD studies and include all interventions for the treatment of OUD including psychosocial and medical treatments of OUD covered by the mandate of the CTN [URL: https://www.drugabuse.gov/about-nida/organization/cctn/clinical-trials-network-ctn].

## Methods/design

In the context of principles of treatment, patient involvement and low administrative burden, and informed by the COnsensus-based Standards for the selection of health Measurement INstruments (COSMIN [27]) and the COMET handbook [28], COS-OUD will be developed following the COS-STAD guideline [29], COS-STAP statement [30], and will be reported following the COS-STAR guideline [31]. The study is registered on the COMET website [URL: http://www.comet-initiative.org/Studies/Details/1579]. The study will use an e-Delphi consensus method with data capture via web-based questionnaires, with final panel results reported anonymously.

### Panel membership and recruitment

The size of a panel for the study will be determined following the principle of inclusivity. Membership of the panel will be open to employed professionals in all 18 CTN nodes who are either clinical addiction practitioners (accredited with a current license as a physician, physician assistant, psychologist, social worker, pharmacist, nurse practitioner, or nurse) or researchers and data administrators. It is anticipated that a panel size of about 40 members will be recruited. In many COS development studies, panel members are not permitted to contribute to subsequent rounds of data collection if they have not contributed to a previous round, but due to the circumstances currently taking place with the COVID-19 pandemic, we plan to relax this approach and allow for a more open process. To minimize attrition, we will clearly set out the importance of full participation and emphasize to members attrition can undermine the degree of consensus in the results. However, we will allow panel members to miss a round and will ask members who leave the study to volunteer a reason as part of efforts to assess possible validity threats to the consensus process [32].

### Review of prior and current CTN OUD trials

As a prelude to the Delphi process, the lead team will undertake a review of past and current CTN OUD trials to assess the current state of measures. This review will be undertaken using the CTN Dissemination Library [URL: http://ctndisseminationlibrary.org/] which has all of the documentary data for the CTN and the parameters will be on treatment studies with OUD outcomes. In addition, members of the expert workgroup will undertake a scoping review to gather potential items for the COS-OUD that will be fed directly into the Delphi process. During this phase, panel members may also nominate specific items during the Delphi protocol.

### Assessing domains of measurement

In addition to specific items that have the potential to serve as elements in the OUD-COS, panel members will be first asked to rank broad domains of measurement (i.e., use per unit time, craving, withdrawal) in the early rounds of reviews. These domains will be used to reflect on the relative importance of major categories of measurement. This exercise will allow the Delphi process to highlight a higher level of conceptual thinking and avoid getting too focused on representation within individual measures. The purpose of doing this before the individual item rounds of questions is to assess whether thinking about specific items changes the perceived importance of categories. Rankings data will be presented to the panel prior to each new round of questions.

### Delphi questionnaire and analysis

We will use REDCap (version 6.18.1) to create web-accessible questionnaires. Each questionnaire will be online for up to 4 weeks with reminder emails sent to panel members every 7 days. In the first round, we will invite panel members to rank the importance of each outcome measure on a 1–9 scale (1–3 rated “not important for inclusion”; 4–6 rated “important but not critical”; 7–9 rated “critical for inclusion”). Percent agreement is the most commonly used method for consensus definition with thresholds ranging from 50 to 97% according to a review of Delphi studies [33]. We will set the consensus definition at 70% or greater [34, 35].

Panel members will see the results in the second round and with eventual consensus defined as 70% (or greater) or more of the respondents scoring an outcome from 7 to 9 and fewer than 15% scoring it 1–3. This approach has an established track record of use and is recommended by the Grading of Recommendations Assessment, Development and Evaluation (GRADE) Working Group for assessing the level of importance about research evidence [https://www.gradeworkinggroup.org]. Should we have three rounds result in a lack of consensus or lack of movement toward it, then we would declare the process will be ended and the varied sides/camps will be reported.

Panel members will be asked to provide recommendations regarding any additions and/or deletions to the list of proposed items, and for any other comments/suggestions. Each questionnaire should take no more than 20–40 min to complete, with the facility to complete it over several sessions and to allow participants to review their answers before the final submission of their responses. De-identified results comprising a narrative summary of findings, comments, and suggestions will be sent to each panel member after the first round. In a second round, ambiguous items or proposals collected from the first round will be compiled (with input from the workgroup as required) before being included in the new round.

Due to COVID-19 challenges for all stakeholders, we plan to handle missing data with a relatively flexible approach. In the event that panel members fail to respond to a single survey, we plan to allow these members to join future rounds of the panel. Individual items that participants skip or opt to not respond to will be re-calculated by using a lower number of panel members thereby accounting for the lack of response.

### Consensus meeting and dissemination plan

Prior to the final determination of the COS-OUD and communication of study results in a peer-reviewed academic journal, we plan to organize a webinar or face-to-face consensus meeting open to CTN nodes, service user representatives, and other key CTN stakeholders to discuss the results of the Delphi process. Depending on the state of travel related to COVID-19, the meeting may be in-person or virtual. Our plan is to have the workgroup leads (Karnik and Marsden) facilitate the meeting. The meeting will be open to all members of the CTN regardless of participation on the eDelphi panel.

## Discussion

This protocol describes a study to develop a COS for OUD-related treatment efficacy and effectiveness trials and allied research. The literature on OUD includes many different outcome measures leading to a lack of clarity on which outcomes are of most importance to researchers and patient stakeholders in terms of understanding experience over the treatment cascade. This in turn has impeded evidence synthesis with missed opportunities to develop pooled research data or to compare results across studies to inform treatment policy and practice.

A previous sustained effort to promote a common approach to the use of outcomes measures has favored a multi-dimensional outcome instrument approach [9]. This study will take an alternative strategy with the goal of identifying a minimum set of outcome items that can be used in all efficacy and effectiveness studies.

### Study status

At the point of manuscript submission, a proposal for the study had been approved, a workgroup had been formed, Institutional Review Board clearance had been received, and the study had been registered with the COMET website. The first phase of this Delphi process is now underway along with the recruitment of the panel.

## Data Availability

Survey data from the eDelphi process will be archived at Rush University for a period of 5 years. Investigators interested in reviewing the survey data directly may do so by contacting staff at the Great Lakes Node of the National Drug Abuse Treatment Clinical Trials Network.
